# Relationship between intraoperative regional cerebral oxygen saturation trends and cognitive decline after total knee replacement: a post-hoc analysis

**DOI:** 10.1186/1471-2253-14-58

**Published:** 2014-07-21

**Authors:** Fátima Salazar, Marta Doñate, Teresa Boget, Ana Bogdanovich, Misericordia Basora, Ferran Torres, Isabel Gracia, Neus Fàbregas

**Affiliations:** 1 Department of Anesthesia, Hospital Clinic de Barcelona, (Universitat de Barcelona), Barcelona, Spain; 2 Department of Neuropsychology, Hospital Clinic de Barcelona, (Universitat de Barcelona), Barcelona, Spain; 3 Statistics & Methodology Support Unit (USEM), IDIBAPS, (Hospital Clinic de Barcelona), Laboratory of Biostatistics & Epidemiology (Universitat Autonoma de Barcelona), Barcelona, Spain

**Keywords:** Cerebral oximetry, Near-infrared spectroscopy, Postoperative memory dysfunction, Postoperative visual-motor coordination and executive function, Postoperative psychological decline

## Abstract

**Background:**

Bilateral regional brain oxygen saturation (rSO_2_) trends, reflecting intraoperative brain oxygen imbalance, could warn of brain dysfunction. Various types of cognitive impairment, such as memory decline, alterations in executive function or subjective complaints, have been described three months after surgery. Our aim was to explore the potential utility of rSO_2_ values as a warning sign for the development of different types of decline in postoperative psychological function.

**Methods:**

Observational post-hoc analysis of data for the patient sample (n = 125) of a previously conducted clinical trial in patients over the age of 65 years undergoing total knee replacement under spinal anesthesia. Demographic, hemodynamic and bilateral rSO_2_ intraoperative values were recorded. An absolute rSO_2_ value of <50% or a reduction of >20% or >25% below baseline were chosen as relevant cutoffs. Composite function test scores were created from baseline to three months for each patient and adjusted for the mean (SD) score changes for a control group (n = 55). Tests were used to assess visual-motor coordination and executive function (VM-EF) (Wechsler Digit Symbol-Coding and Visual Reproduction, Trail Making Test) and memory (Auditory Verbal Learning, Wechsler Memory Scale); scales were used to assess psychological symptoms.

**Results:**

We observed no differences in baseline rSO_2_ values; rSO_2_ decreased significantly in all patients during surgery (P < 0.0001). Seventy-five patients (60%) had no sign of cognitive decline or psychological symptoms. Twenty-one patients (16.8%) had memory decline, 3 (2.4%) had VM-EF decline, and 33 (26.4%) had psychological symptoms. Left and right rSO_2_ values were asymmetric in patients who had memory decline (mean [SD] left-right ratio of 95.03 [8.51] vs 101.29 [6.7] for patients with no changes, P = 0.0012). The mean right-left difference in rSO_2_ was also significant in these patients (-2.87% [4.73%], lower on the right, P = 0.0034).

**Conclusions:**

Detection of a trend to asymmetry in rSO_2_ values can warn of possible postoperative onset of memory decline. Psychological symptoms and memory decline were common three months after knee replacement in our patients over the age of 65 years.

## Background

Non-invasive recording of regional cerebral oxygen saturation (rSO_2_) may facilitate intraoperative continuous monitoring of changes in oxygenation and cerebral hemodynamics [1-4]. Casati et al. [5] hypothesized that controlling intraoperative rSO_2_ may help attenuate the possible effects of brain hypoxia on cognitive function. These and other authors [6-9] have found rSO_2_ reduction to be significantly correlated with decline, specifically postoperative cognitive dysfunction (POCD) after abdominal and cardiac surgery under general anesthesia. A recent meta-analysis, however, did not find evidence to support this correlation in cardiac surgery [10].

Individuals older than 60 years had higher risk for POCD after major non-cardiac surgery in a study by Monk et al. [11], and we also saw increased risk in a trial enrolling patients who underwent knee replacement under spinal anesthesia [12]. When Price et al. [13] analyzed a subgroup of the non-cardiac surgical patients of Monk et al., they were able to identify different types of decline, finding memory impairment in 13.6%, executive function impairment in 8.4%, and a combination of both in 2.9%. Since we had information available on both rSO_2_ trends and specific types of decline for patients in our earlier study [12], we hypothesized that a post-hoc analysis of the data might confirm the findings of Price et al. and also allow us to explore a possible relationship between type of cognitive decline and either right-brain or left-brain desaturation. Our psychological assessments encompassed memory, visual-motor coordination and executive function (VM-EF) as well as subjective psychological complaints of memory loss, inability to concentrate, anxiety, and depression. Thus, some of the neuropsychological tests utilized evaluated functions predominantly related to one hemisphere or the other.

We designed the present post-hoc analysis of rSO_2_ trends in the hope of finding more specific information about the development of cognitive decline. Our aim was to explore the utility of rSO_2_ values for warning of different types of decline.

## Methods

This post-hoc analysis of the patient sample of a previously conducted clinical trial [12], was approved by the Ethics Committee of Hospital Clinic, Barcelona, Spain, on 22 June 2005 (Ref. 2569). We recruited patients of both sexes over the age of 65 years who were scheduled for total knee replacement under spinal anesthesia, applying the following exclusion criteria: a history of central nervous system disorder, recognized abnormalities in supraaortic branches, prior neurosurgery or use of medications acting on the central nervous system (tranquilizers, antidepressants, etc.). All patients were in American Society of Anesthesiologists class 1 or 2 and voluntarily signed their informed consent before enrollment.

Bilateral rSO_2_ sensors were placed on the right and left sides of the forehead prior to spinal anesthesia. Values were continuously measured using near-infrared spectroscopy (INVOS 4100; Somanetics Inc, Troy, MI, USA). We analyzed rSO_2_ behavior throughout the procedure in all patients. Oxygen desaturation cutoffs were defined as established in the literature in three ways, as follows: an absolute rSO_2_ reading of <50% or a reduction of >20% or >25% of the patient’s baseline rSO_2_[5,6,9,14-16] lasting ≥15 seconds [5,16]. Percentage desaturation from baseline was recorded in order to normalize rSO_2_ data, following the method of Mille et al. [17] so that we could compare rSO_2_ changes in any phase of the study between patients, given that inter-subject variability in rSO_2_ values has been reported [3,18].

To evaluate learning and memory, which are predominantly left hemisphere functions, we used two instruments: the Auditory Verbal Learning Test [19] and the Wechsler Memory Scale, third edition [20]. To evaluate VM-EF performance, which reflects mainly right brain hemisphere activity, the following tests were included: the Digit Symbol-Coding [21] and Visual Reproduction subtests of the Wechsler Adult Intelligence Scale, third edition [20] and parts A and B of the Trail Making Test for sustained attention and mental flexibility, respectively [22].

Following Price et al. [13], we used reliable change scores to assess cognitive change from baseline to three months for each patient. These scores were calculated for each neuropsychological assessment by first computing the difference from baseline to three months for each subject, then computing the mean and SD of the change scores for a control group (55 controls, as described previously [12]), and finally standardizing the change score for the study patients by subtracting the control group’s mean and dividing the result by the control group’s SD. In this way, the scores of the controls were used to adjust for any learning effect in the study patients in the surgical group. We next formed composite scores for the VM-EF and memory indices. The severity of cognitive change was graded as mild, moderate, or severe based on the SDs of these composite scores. Mild decline was defined as a change of 1 SD, moderate as a change of 1.5 SD, and severe as ≥2 SDs.

Psychological state was also evaluated with instruments to measure symptoms of anxiety, depression, subjective perception, and neurosis, as follows: the Hospital Anxiety and Depression Scale [23], a health-related quality of life scale [24], and the neuroticism scale from of a modified version of the Eysenck Personality Questionnaire [25] (Appendix 1).

To test the hypothesis that desaturation might be related to specific neuropsychological test results reflecting functions controlled from different parts of the brain, we divided the study population into four groups according to their three-month status: 1) patients with no signs of postoperative dysfunction detected by any neuropsychological test (no-change group), 2) patients with memory decline, 3) patients with VM-EF deficit, and 4) patients with psychological symptoms. We compared right and left rSO_2_ trends between the no-change group and each of the other groups.

Patients were administered combined spinal–epidural anesthesia with 10 mg of 0.5% bupivacaine plus 10 mg of fentanyl. Midazolam (1–2 mg) was also administered for sedation. Postoperative patient-controlled analgesia was provided by means of a continuous infusion of 0.2% ropivacaine plus 2 mcg/ml of fentanyl. Dexketoprofen was given at a dosage of 50 mg/8 h. Paracetamol was given as a rescue analgesic (1 g/8 h) if the patient assessed pain >3 on a visual analogue scale. This analgesic schedule was maintained for 48 hours.

Patient demographic characteristics were on record, as were durations of surgery, tourniquet induced ischemia, and motor block. Other variables recorded included blood loss, hemoglobin and transfusion requirements until discharge and hospital length of stay. C-reactive protein (CRP) levels were measured at hospital admission. Hemodynamic variables (blood pressure and heart rate), respiratory variables (respiratory rate, capnography, and oxygen saturation by pulse oximetry), and tympanic temperature were also measured.

All these data were recorded in the operating room for analysis at the following times: T1, prior to induction (baseline); T2, 10 min after spinal puncture; T3, just before inflating the tourniquet; T4-T6, 5 min, 15 min and 30 min, respectively, after inflating the tourniquet; T7, just before deflating the tourniquet; T8-T11, during reperfusion at 5 min, 10 min, 15 min, and at the end of surgery, respectively, after deflating the tourniquet; T12 and T13, postoperatively at 15 min and 30 min; and T14-T18, postoperatively every half hour for 3 hours (at 60, 90, 120, 150, and 180 min) during recovery from the motor block. These data sampling times were selected to coincide with expected periods of greater hemodynamic change during the surgical procedure and recovery. After induction of spinal anesthesia, nasal prongs were set in place to deliver 3 l/min of oxygen during surgery; the prongs were left in place until the next-to-last measurement in the recovery area.

### Statistical analysis

This post-hoc analysis used data for a patient sample (n = 125) from a previously conducted clinical trial [12]; data were included for all patients who completed the neuropsychological tests three months after surgery.

The Fisher exact test was used to compare categorical values. To compare continuous variables recorded at a specific moment we used the t-test, and for longitudinal analysis we used a mixed models analysis of variance for repeated measurements, setting the (co)variance matrix to unstructured. To study correlations we used the Pearson method. Analyses were performed using SAS software, version 9.2 (SAS Institute Inc., Cary, NC, USA). The level of significance was established at P < 0.05 in two-sided tests.

## Results

Demographic characteristics (including sex and age, mean times, duration of hospital stay, and blood loss or transfusion requirements) are shown in Table 1. The control group was similar in gender (27 men, 28 women) and age (mean [SD] age, 74 [6.26] years).

**Table 1:** Patients’ baseline demographic characteristics, laboratory findings, perioperative blood parameters and durations

Gender, male/female (n)	29/96
Age (yr)	72.8 (4.5)
Weight (kg)	74.3 (11.8)
Height (cm)	157.9 (7.5)
ASA score (1/2) (%)	21.6/78.4
Tourniquet time (min)	61.1 (16.5)
Duration of surgery (min)	89.4 (20.3)
Motor block recovery time (min)	243.3 (48.5)
Baseline Hb (g/dL)	13.3 (1.1)
Day 4 Hb (g/dL)	9.2 (0.8)
Blood loss (% of total volume)	47.3 (17.7)
Transfused patients (%)	47.2
CRP >0.8 mg/dl (n [%])	32 (25.6%)
Length of hospital stay (d)	5.2 (1.1)

Results are presented as absolute number, percentage of 125 patients, or means (SD) in the units indicated.

ASA = American Society of Anesthesiologists.

CRP = C-reactive protein.

Hb = hemoglobin.

**Figure 1:**
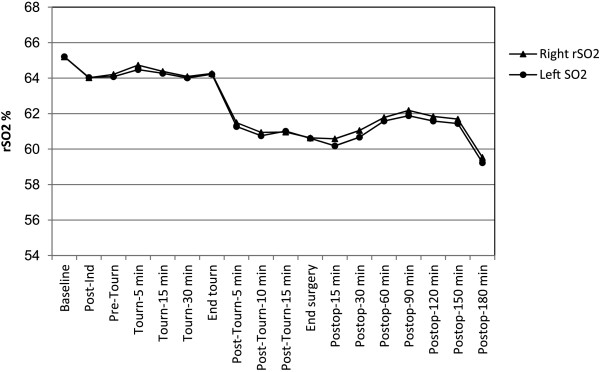
**Least mean squares estimates of regional cerebral oxygen saturation (rSO**_**2**_**) in each hemisphere over time for all patients (n = 125).** rSO_2_ values for both hemispheres were seen to decrease over the course of study (P < 0.0001). The largest rSO_2_ decline coincided with tourniquet deflation and reperfusion of the extremity. Three hours after surgery rSO_2_ had not recovered to baseline (59.46% [95% CI, 58.25%–60.66%] and 59.20% [95% CI, 58.14%–60.27%] (P < 0.001) for right and left hemispheres respectively. CI = confidence interval. Ind = induction. Tourn = tourniquet. Postop = postoperative.

The mean (SD) baseline absolute rSO_2_ values in the study group were 65% (7.3%) (range, 44%–87%) for the right hemisphere and 65% (6%) (range, 47%-80%) for the left hemisphere. rSO_2_ values for both hemispheres were seen to decrease over the course of study (P < 0.0001) (Figure 1), and the largest declines coincided with cuff deflation and reperfusion of the extremity. The mean right and left absolute rSO_2_ values three hours after surgery (59.46% [95% confidence interval, 58.25%–60.66%] and 59.20% [95% confidence interval, 58.14%–60.27%], respectively) had not recovered to baseline levels (P < 0.001). No correlations between changes in rSO_2_ and blood loss, hemodynamic or other respiratory variables were found. Baseline absolute rSO_2_ values of <50% were observed only in two patients (in one hemisphere).

Absolute rSO_2_ readings of <50% were observed throughout the procedure (Table 2). The baseline rSO_2_ readings of patients who reached these low values were significantly lower (P < 0.0001) than those of patients without such severe desaturation, and they also had greater relative reductions from baseline in both hemispheres. Reductions of >20% or >25% below the patient’s baseline rSO_2_ were also observed throughout the procedure (Table 2).

**Table 2:** **Absolute rSO**_**2 **_**readings of <50% or a reduction of >20% or >25% below the patient’s baseline rSO**_**2 **_**observed during the procedure**

**rSO**_**2**_	**No. of patients**	**Baseline rSO**_**2 **_**values**		
	**n (% of 125)**	**Mean (SD)**	**Range**	P*
Right hemisphere				
Absolute value				
≥50%	99 (79.2)	66.7% (6.6%)	53%–78%	<0.0001
<50%	26 (20.8)	59.1% (6.5%)	44%–78%	
Reduction from baseline				
>20%	22 (17.6)			
>25%	11 (8.8)			
Left hemisphere				
Absolute values				
≥50%	103 (82.4)	66.4% (5.5%)	53%–80%	<0.0001
<50%	22 (17.6%)	59.9% (5.3%)	47%–70%	
Reduction from baseline				
>20%	20 (16)			
>25%	8 (6.4)			

*Comparison of baseline rSO_2_ of patients who reached an absolute rSO_2_ reading <50% and those who did not.

rSO_2_ = regional cerebral oxygen saturation.

Three months after surgery 75 patients (60%) had no sign of cognitive decline or psychological symptoms. Twenty-one patients (16.8%) had memory decline (15 mild, 4 moderate, 2 severe), three (2.4%) had VM-EF deficits (1 mild, 1 moderate, 1 severe), and only one (0.8%) showed a combined decline. Thirty-three patients (26.4%) had acquired psychological symptoms (depression, anxiety, or subjective complaints about memory or concentration) in isolation or in combination (P = 0.017). These symptoms were associated with measures of cognitive decline in eight patients (6.4%) (six patients with memory deficit, one patient with VM-EF deficit, and one patient with both types of decline). We excluded these eight patients from further analysis because they had a mixed pattern of memory decline, psychological symptoms or VM-EF decline.

**Figure 2:**
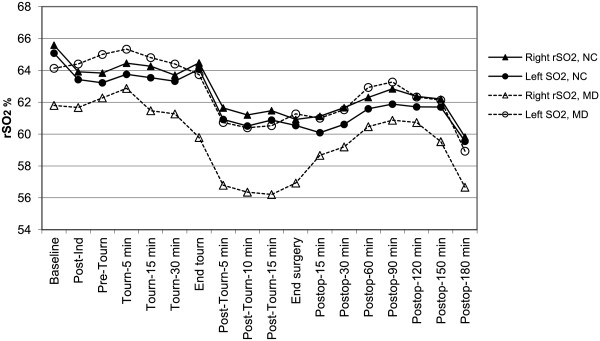
**Least mean squares estimates of regional cerebral oxygen saturation (rSO**_**2**_**) in each hemisphere over time in patients with no cognitive changes (NC) and in patients who developed memory decline (MD).** Both right and left rSO2 values decreased over the course of the procedure in both groups (P < 0.001). Patients with MD showed significant right-left asymmetry in rSO_2_ values (P = 0.0012) as well as a significant mean right-left rSO_2_ difference (P = 0.0034). Ind = induction. Tourn = tourniquet. Postop = postoperative.

We observed no differences in the baseline rSO_2_ values of patients with only memory decline (15 patients) at three months and those with no change. Nonetheless, patients with memory decline showed greater asymmetry between left and right rSO_2_ values over the course of the study, evident in a significantly lower right-left ratio of rSO_2_ mean values; their mean difference between the right and left rSO_2_ values was also greater (Table 3) (range in difference, 1%–10.94%). Finally, the percentage of memory-decline patients who had right rSO_2_ reductions of >25% from baseline was higher than in the group with no cognitive changes (P = 0.0226). Figure 2 compares the right and left rSO_2_ values of patients with memory decline and without changes.

**Table 3:** Clinical data in patients without neuropsychological changes compared to data for patients with memory decline or psychological symptoms

	**No change (n = 75)**	**Memory decline (n = 15)**	P*	**Psychological symptoms (n = 25)**	P*
Gender, male/female (%)	20/80	40/60	0.1756	24/76	0.7796
ASA score 1/2 (%)	21.3/78.7	13.3/86.7	0.7291	24/76	>0.999
Baseline right rSO_2_	65.71% (7.8%)	61.8% (9.2%)	0.0861	65.56% (6.8%)	0.9746
Baseline left rSO_2_	65.19% (6.2%)	64.13% (6.5%)	0.4149	65.64% (4.4%)	0.6908
Final right rSO_2_	59.99% (6.7%)	56.67% (6.8%)	0.1201	59.50% (6.7%)	0.7771
Final left rSO_2_	59.63% (6.8%)	58.93% (6.8%)	0.7311	58.29% (6.8%)	0.4201
Right rSO_2_ < 50% (n [%])	13 (17.3)	5 (33.3)	0.2845	7 (28)	0.3889
Right desaturation >20% (n [%])	14 (18.7)	4 (26.7)	0.7280	4 (16)	>0.999
Right desaturation >25% (n [%])	4 (5.3)	4 (26.7)	0.0226	3 (12)	0.3642
Left rSO_2_ < 50% (n [%])	13 (17.3)	1 (6.7)	0.4507	6 (24)	0.5612
Left desaturation >20% (n [%])	13 (17.3)	0 (0)	0.1171	6 (24)	0.5612
Left desaturation >25% (n [%])	5 (6.7)	0 (0)	0.5906	3 (12)	0.6771
Right-left ratio of rSO_2_ (n [%])	101.29 (6.17)	95.03 (8.51)	0.0005	100.46 (8.11)	0.2678
Mean right-left rSO_2_ difference	0.76 (4.16)	-2.87 (4.73)	0.0008	0.15 (4.66)	0.3498
Minimum temperature in°C	35.03 (0.78)	35.01 (0.75)	0.8163	34.88 (0.78)	0.4275
Mean temperature in °C	35.57 (0.72)	35.48 (0.67)	0.5459	35.44 (0.70)	0.4828
Blood loss (% of total volume)	47.33 (14.33)	38.89 (10.59)	0.0333	46.06 (21.43)	0.5950
Transfused patients (n [%])	37 (49.3)	3 (20)	0.0456	13 (52)	>0.999
No. of blood units transfused	0.81 (0.95)	0.33 (0.72)	0.0607	1 (1.08)	0.4638
Vasoactive drugs (atropine or ephedrine) (n [%])	20 (26.7)	4 (26.7)	>0.999	9 (36)	0.4438
Minimum perioperative Hb (g/dL)	8.72 (0.78)	9.19 (0.92)	0.0502	8.83 (1.19)	0.8146
Hb at discharge (g/dL)	9.09 (0.10)	9.67 (0.13)	0.0111	9.50 (0.18)	0.0491
CRP >0.8 mg/dl (n [%])	14 (18.6)	5 (33.3)	0.2531	10 (40)	0.0603

Data are presented as means (SD), unless otherwise stated on each row as percentage of patients or number and percentage of patients.

*Group with memory decline vs group with no changes, or group with psychological symptoms vs group with no changes.

ASA = American Society of Anesthesiologists.

CRP = C-reactive protein.

Hb = hemoglobin.

rSO_2_ = regional oxygen saturation.

**Figure 3:**
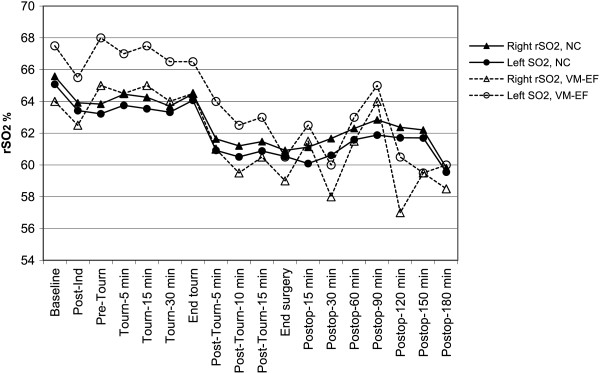
**Least mean squares estimates of regional cerebral oxygen saturation (rSO**_**2**_**) in each hemisphere over time in patients with no cognitive changes (NC) and in the two patients who developed only visual-motor and executive function (VM-EF) decline.** Ind = induction. Tourn = tourniquet. Postop = postoperative.

Only two patients had exclusively VM-EF decline, and their baseline rSO_2_ values were similar to those of other patients. Neither of these two patients had rSO_2_ levels <50% or reached desaturation levels of >20% over baseline. Over the course of their procedures, however, these patients did have fluctuating bilateral rSO_2_ decreases (Figure 3) that were statistically significant (P < 0.001).

**Figure 4:**
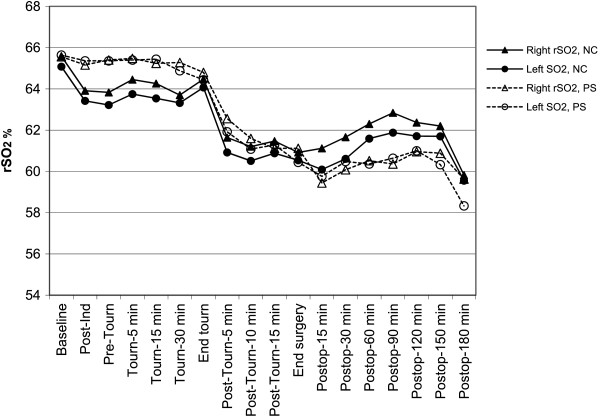
**Least mean squares estimates of regional cerebral oxygen saturation (rSO**_**2**_**) in each hemisphere over time in patients with no cognitive changes (NC) and in the patients who showed psychological symptoms (PS).** The trend in rSO_2_ desaturation over the course of surgery was significant (P < 0.001) in both groups; the between-group differences or between-hemisphere differences were not significant. Ind = induction. Tourn = tourniquet. Postop = postoperative.

Patients with psychological symptoms had baseline rSO_2_ values that were similar to those of patients with no change. However, they were more likely to reach absolute rSO_2_ values <50% in both hemispheres and have desaturation >20% from baseline in the left hemisphere than patients who were change-free (Figure 4). Patients reporting psychological symptoms also tended to have higher CRP plasma levels (P = 0.0603) (Table 3).

The only patient with combined decline (memory decline, altered VM-EF, and psychological symptoms) showed strikingly asymmetric left-right rSO_2_ values throughout the procedure (range of change, 9%–11%); the left-hemisphere values were lower.

Blood loss differed between patients with no change and those with memory deficits (P = 0.0330), and more patients in the group with no change received transfusions than in the group with memory decline (P = 0.0456). Hemoglobin values at discharge from hospital were lower in patients without memory (P = 0.011) or psychological (P = 0.049) changes (Table 3).

## Discussion

Unlike studies that have compared perioperative rSO_2_ by focusing on the lowest value reached, regardless of hemisphere [5-7,26], we investigated the changes that occurred in each hemisphere independently and tested the hypothesis that they might be related to change detected by neuropsychological tests of functions governed by the right or left hemisphere.

A decrease in the right rSO_2_ of >25% from baseline was observed throughout the procedure in patients with memory decline and was significantly greater than the rSO_2_ decrease seen in patients without any cognitive impairment. Therefore, we emphasize this between-hemisphere asymmetry in rSO_2_ value changes we found in patients with memory decline. As far as we know this trend has not been previously described.

The two patients with exclusively VM-EF impairment in our study showed remarkable fluctuations in rSO_2_ values. Similarly, our only patient with combined impairment (simultaneously impaired memory, VM-EF, and subjective decline) had highly asymmetric right-left rSO_2_ values throughout the procedure. In this particular patient, left values were lower. We suggest, that marked asymmetry in rSO_2_ values may reflect an imbalance in brain oxygen consumption or delivery. Based on our small post-hoc analysis, we do not know what asymmetry cut-off might be reflected in specific types of cognitive decline.

Our findings indicate that impaired memory is evident three months after knee replace surgery in one out of six patients operated under spinal anesthesia. Moreover, we note that subjective psychological symptoms were detected at that time in patients without associated cognitive decline.

In our series, more patients had memory impairment (16.8%) than VM-EF impairment (2.4%) or combined decline (0.8%), consistent with the findings of Price et al. [13], who reported that 13.6% had detectable memory decline, 8.4% had executive function decline, and 2.9% a combination of both. The slight differences between their study and ours can be explained by population variability. We had designed our initial study [12] to have a population of patients with similar characteristics who were in relatively good health (ASA 1–2). They were operated on early in the morning [27,28] and were subject to the same protocols for anesthesia and postoperative care. We excluded patients who underwent revision knee replacements or any other operation before the third neuropsychological test battery was administered. The different types of decline that we and Price et al. observed lead us to suggest that left hemisphere, where memory is predominantly processed, may be more clinically vulnerable than the right hemisphere, where activity related to VM-EF predominates. This hypothesis requires further prospective testing, however.

Thirty-three of our patients (26.4%) reported psychological symptoms on subjective scales at the three month follow-up. We find it interesting that for most of these patients no memory or VM-EF decline was detected by the instruments used. This finding of subjective complaints of memory and concentration difficulty unrelated to the appearance of POCD detected by specific instruments has also been reported by other authors [11,29-31]; in the study of Dijkistra et al. [31] the subjective impressions were still present at six-month follow-up in 17% of the patients.

Maze [32] suggested that the cognitive decline seen in the elderly after surgery might reflect an exacerbation of aging processes, further hypothesizing that a context of reduced cognitive reserve will favor the development of POCD. In Maze’s opinion surgery or anesthesia can accelerate the mechanisms leading to age-related cognitive decline, as oxidative stress has been mentioned as a likely cause of neurodegeneration in the elderly. Within the aging brain, there is a proinflammatory phenotype with up-regulation of markers such as interleukin 6 and CRP. In our study, presurgical CRP values did tend to be higher in the patients with memory decline or subjective symptoms than in patients without any type of dysfunction. The differences were not statistically significant, however, possibly because of our small sample size.

Complaints of memory loss are often the first and main warning of decline in cognitive reserve [33]. Baseline cerebral oxygen saturation diminishes with age, as does the response of oxygen saturation during activity in the brain hemispheres [34,35]. Patients presenting only psychological symptom complaints had no asymmetry or fluctuations in rSO_2_ trends. However, more patients with subjective complaints reached absolute bilateral rSO_2_ values <50% and left-sided desaturation of >20% below baseline than in the group with no changes.

Heringlake et al. [36] found that patients with low baseline rSO_2_ values that do not increase with oxygen administration have higher rates of postoperative morbidity and mortality than patients who do respond to oxygen. In our study, patients with the lowest baseline rSO_2_ values were at higher risk of further desaturation and were those who were most likely to reach an absolute reading of <50%, the level that seems to be indicative of cerebral ischemia and risk of neurologic complications [3,14,37]. Baseline low oxygen saturation might reflect the inability of cerebral tissues to absorb more oxygen in response to diminished supply [3]. Increasing oxygen deliver is one of the measures intended to improve cerebral desaturation; however, we did not explore the response of rSO_2_ to oxygen administration in our initial trial [12], so these data were not available.

The first limitation of this post-hoc analysis is that the patient cohort was originally recruited for a randomized trial in the setting of a specific intervention [12]. However, the patients in the two groups had similar baseline characteristics and there was no clinically or statistically significant effect of temperature on the assessment of memory decline and psychological symptoms. Therefore, we felt that pooling the data for those randomized patients for use in this post-hoc analysis would be justified. Another limitation was that we recorded the baseline rSO_2_ values before the patients were on supplemental oxygen. The incidence of relative desaturations would probably have been higher if baseline readings were taken with supplemental oxygen in place. However, this decision was consistent with the use of our use of the preanesthetic reading as the baseline, as in other studies [5,6,9,38]. Only Murkin and Arango [4] administered oxygen before the baseline reading.

Surprisingly, hemoglobin values at hospital discharge were lower in patients without changes even though more patients received blood transfusion in these patients without impairment than in those with memory decline. We do not know the significance of this observation.

## Conclusions

On the basis of our results we can suggest that the detection of asymmetry in perioperatively rSO_2_ values should alert caregivers to the possibility of postoperative onset of memory decline. We saw impaired memory in almost 17% of our patients and almost 25% complained of psychological symptoms three months after total knee replacement under spinal anesthesia.

## Appendix 1

### Neurocognitive evaluation

*Vocabulary* (of the Wechsler Adult Intelligence Scale, version III [WAIS III]) [21]. This vocabulary subtest assesses words in long-term memory, giving a score that reflects intellectual level; performed only once, at baseline.

#### Learning and verbal memory

These tasks require language and function mainly depends on the dominant hemisphere (usually the left).

• Auditory Verbal Learning [19]: the patient is asked to remember as many words as possible from a list of 15. Immediate recall is requested and the average of five trials is recorded; a score for delayed, or medium-term memory, is also recorded.

• Memory Logic I-II (of the Wechsler Memory Scale, third edition [WMS III]) [20]. These subtests assess immediate memory recall of a prose text (subtest I) and medium-term memory recall (subtest II).

#### Visual-motor coordination and executive function

These tests involve visual-motor tracking, visual conceptual and visual organization and memory; these functions are predominantly related to the non-dominant hemisphere (usually the right).

• Digit Symbol-Coding (WAIS III) [21], tasks that make fine motor demands and assesses visual-manual coordination; it includes a speed component that assesses new learning ability, sustained attention, processing speed, and psychomotor speed.

• Trail Making Test (TMT-A and TMT-B) [22], tasks that evaluate motor and spatial organization skills in response to stimuli, performance speed, and sustained attention (TMT-A) as well as mental flexibility (TMT-B).

• Visual Reproduction I–II (WMS III) [20], tasks in which both immediate (I) and delayed (II) memory of visual content are tested.

### Clinical examination of psychological state and dysfunction

#### Subjective complaints about memory or concentration

The Cognitive Functioning Scale of the Spanish version of the Quality of Life in Epilepsy Inventory (version 1.0) adapted and validated for use in the Spanish population [24].

#### Neuroticism

An adapted Spanish version of the Eysenck Personality Questionnaire, validated for use in the Spanish population [25].

#### Anxiety and depression

Hospital Anxiety and Depression [23].

## Abbreviations

CRP: C-reactive protein; POCD: Postoperative cognitive dysfunction; rSO_2_: Regional cerebral oxygen saturation; VM-EF: Visual-motor - executive function; ASA: American Society of Anesthesiologists; Hb: Hemoglobin; MD: Memory decline; NC: No changes; Postop: Postoperative; PS: Psychological symptoms; Tourn: Tourniquet.

## Competing interests

The authors declare that they have no competing interests.

## Authors’ contributions

FS conceived the study, participated in its design and coordination, recruited and informed the patients, collected and interpreted data, and helped to draft the manuscript. MD and TB participated in design and data collection, analysis and interpretation. AB, MB, and IG recruited and informed the patients and participated in data collection. FT made substantial contributions to the statistical design, undertook the statistical analysis, interpreted the data, and helped to critically revise the manuscript. NF participated in the design and has been involved in drafting and revising the manuscript critically for important intellectual content; she has given final approval of the version to be published. All authors have read and approved the final manuscript.

## Pre-publication history

The pre-publication history for this paper can be accessed here:

http://www.biomedcentral.com/1471-2253/14/58/prepub
